# The roles of dystroglycan in the nervous system: insights from animal models of muscular dystrophy

**DOI:** 10.1242/dmm.035931

**Published:** 2018-12-19

**Authors:** Alec R. Nickolls, Carsten G. Bönnemann

**Affiliations:** 1 National Institute of Neurological Disorders and Stroke, National Institutes of Health, Bethesda, MD 20892, USA; 2 Department of Neuroscience, Brown University, Providence, RI 02912, USA

**Keywords:** Muscular dystrophy, Brain development, Dystroglycan, Animal models

## Abstract

Dystroglycan is a cell membrane protein that binds to the extracellular matrix in a variety of mammalian tissues. The α-subunit of dystroglycan (αDG) is heavily glycosylated, including a special O-mannosyl glycoepitope, relying upon this unique glycosylation to bind its matrix ligands. A distinct group of muscular dystrophies results from specific hypoglycosylation of αDG, and they are frequently associated with central nervous system involvement, ranging from profound brain malformation to intellectual disability without evident morphological defects. There is an expanding literature addressing the function of αDG in the nervous system, with recent reports demonstrating important roles in brain development and in the maintenance of neuronal synapses. Much of these data are derived from an increasingly rich array of experimental animal models. This Review aims to synthesize the information from such diverse models, formulating an up-to-date understanding about the various functions of αDG in neurons and glia of the central and peripheral nervous systems. Where possible, we integrate these data with our knowledge of the human disorders to promote translation from basic mechanistic findings to clinical therapies that take the neural phenotypes into account.

## Abstract (precis)

**Summary:** Dystroglycan is a ubiquitous matrix receptor linked to brain and muscle disease. Unraveling the functions of this protein will inform basic and translational research on neural development and muscular dystrophies.

## Introduction

Dystroglycan is a receptor of extracellular matrix (ECM) proteins in many developing and adult mammalian tissues (Ibraghimov-Beskrovnaya et al., 1993; Blaeser et al., 2018), and is composed of two protein subunits translated from a single mRNA transcript of the *DAG1* gene (Ibraghimov-Beskrovnaya et al., 1992). The α-subunit, designated as α-dystroglycan (αDG), resides at the outer surface of the plasma membrane, where it shares a tight noncovalent bond with the membrane-spanning β-subunit (βDG) (Holt et al., 2000; Akhavan et al., 2008). The intracellular domain of βDG interacts with cytosolic proteins, most notably those of the dystrophin family (Xin et al., 2000; Palmieri et al., 2017). Together, αDG, βDG and dystrophin represent the core functional unit of the dystrophin-glycoprotein complex, physically linking ECM and cytoskeletal elements (see Box 1 for an overview of dystroglycan structure and interactions).

**Box 1. Structure and interactions of dystroglycan**

The *DAG1* gene is transcribed to an mRNA containing a single open reading frame encoding both αDG and βDG (Ibraghimov-Beskrovnaya et al., 1993). The *DAG1* mRNA transcript is translated to a precursor polypeptide that is subsequently cleaved by an unidentified enzyme to generate the αDG and βDG proteins (Holt et al., 2000). αDG consists of a central mucin-like domain flanked by two globular domains (Brancaccio et al., 1995). The C-terminal globular domain is non-covalently linked to βDG at the cell surface, with βDG containing a transmembrane domain and a cytoplasmic C-terminus (see schematic below).

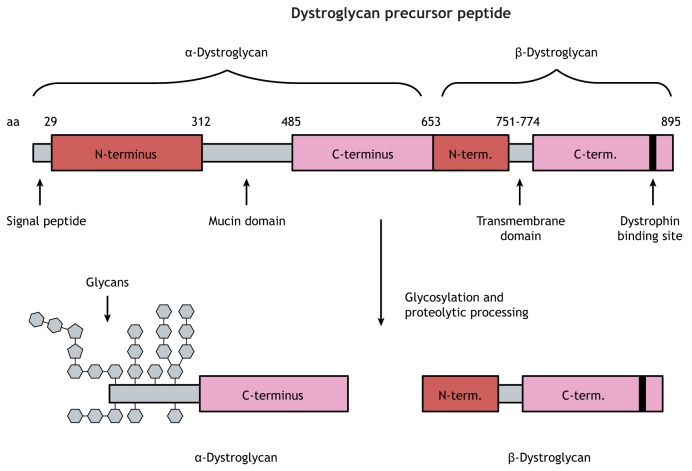

aa, amino acid.

The N-terminal globular domain of αDG is critical for enzymatic recognition and post-translational processing of the protein in the endoplasmic reticulum and Golgi apparatus (Kanagawa et al., 2004). However, the N-terminus is ultimately removed in the Golgi apparatus, and this region is not directly involved in αDG function (Brancaccio et al., 1997). The central mucin-like domain of αDG receives abundant post-translational modification in the form of glycosylation, which is required for interaction between αDG and its ligands.

αDG binds to extracellular proteins, including laminins, agrin and perlecan, in the muscle and brain microenvironment (Ibraghimov-Beskrovnaya et al., 1992; Bowe et al., 1994; Campanelli et al., 1994; Gee et al., 1994; Sugiyama et al., 1994; Peng et al., 1998). Similarly, αDG interacts with slit proteins in the spinal cord, neurexin proteins in the brain and pikachurin in the retina (Sugita et al., 2001; Sato et al., 2008; Wright et al., 2012). These interactions have various structural and functional consequences in their respective tissues, as discussed in this Review. Interestingly, brain αDG has reduced glycosylation and ligand binding affinity compared with that of muscle αDG, but the physiological implication of these differences is unknown (Smalheiser and Kim, 1995; Gesemann et al., 1998; Leschziner et al., 2000).

The expression of this protein complex is widespread; αDG is found in cells of the skeletal muscle, nervous system, digestive tract, kidney, skin and reproductive organs (Durbeej et al., 1998). Many functions have been ascribed to αDG, depending on developmental and cell-specific contexts. αDG participates in basement membrane formation (see Box 2 for a glossary of terms) and in signal transduction from the ECM (Gracida-Jiménez et al., 2017). Further, through physical anchorage with the surrounding matrix, αDG protects muscle cell membranes against contraction-induced damage (Han et al., 2009).

**Box 2. Glossary**

**Agrin:** a proteoglycan secreted by nerve terminals that binds to MuSK and αDG on the postsynaptic muscle membrane. Agrin is the main instructive secreted signal for neuromuscular junction formation.

**Basement membrane:** compact sheets of polymerized matrix proteins, generally composed of laminin, collagen, perlecan and nidogen proteins. Basement membranes can be divided into three layers based on electron microscopy: (1) an electron-sparse ‘lamina lucida’ at the cell surface made of the cell-binding long arm of laminin, (2) an overlying ‘lamina densa’ of type IV collagen, perlecan, nidogen and the crosslinking shorter arms of laminin, (3) and beyond this a ‘lamina reticularis’ of fibrillar collagens. This molecular lattice forms the periphery of many organs, serves to anchor individual cells and provides a framework for tissue structure in all metazoan organisms.

**Cobblestone lissencephaly:** a developmental condition characterized by an unusually smooth brain surface with abnormally formed gyri resembling a ‘cobbled’ exterior, also referred to as lissencephaly type II. It results from over-migration of neurons into the subarachnoid space, resulting in the finely cobbled smooth surface with underlying dense folding or gyri resembling polymicrogyria.

**Cre-driver line:** a mouse strain genetically engineered with a Cre recombinase gene driven by a promoter of choice. When crossed with a strain harboring strategically placed loxP sites in a gene of interest (floxed exons), this will result in temporal and cell-type-selective gene knockout (termed the Cre/*lox* system).

***DAG1*:** the gene encoding αDG and βDG, which are transcribed and translated as one and cleaved post-translationally.

**Electroretinogram (ERG):** an electrode measurement of signaling between photoreceptors and their downstream bipolar and ganglion cells in the retina. On the ERG trace, the first deflection is the a-wave, representing photoreceptor activity, and the second deflection is the b-wave, mediated by ganglion and bipolar cells.

**Embryoid body:** a culture of embryonic stem cells in spherical aggregates that differentiate an inner epiblast-like core and an extraembryonic endoderm-like periphery. Between these two cell layers forms a basement membrane reminiscent of that found between the epiblast and primitive endoderm of the pre-gastrulation mammalian embryo.

**Glycosylation:** the post-translational process of adding carbohydrate chains, or glycans, to proteins by glycosyltransferase enzymes of the endoplasmic reticulum and Golgi apparatus. These sugars often mediate protein folding or protein-protein interactions. A form of glycosylation on αDG that can be disturbed in the α-dystroglycanopathies is referred to as O-mannosylation, indicating the molecular bond and first sugar added.

**Hydrocephaly:** a condition of increased cerebrospinal fluid volume in the brain that leads to expansion of the ventricles and often the skull.

**Hypoglycosylation:** a reduced number of glycans on a glycosylated protein. This is detected by a reduction in the protein's molecular mass and, in the case of αDG, it is concomitant with a reduced ability to bind extracellular ligands such as laminin.

**Laminins:** massive cross-shaped heterotrimeric extracellular matrix proteins that bind to cell surface αDG and integrin. Along with type IV collagen, laminins are major constituents of basement membranes. They are typically composed of one heavy α chain and β and γ light chains. Their nomenclature follows the number for the α, β and γ chain, so that a heterotrimer composed of α2, β1 and γ1 would be designated as laminin 211 or lm211.

***Large*:** the gene encoding like-acetylglucosaminyltransferase (also known as Large), the final bifunctional glycosyltransferase directly responsible for synthesizing matriglycans on αDG.

**LG domains:** protein structures of the laminin globular domain family. They are commonly found in extracellular proteins and are involved in protein-protein interaction.

**Matriglycan:** a unique glycan consisting of repeating disaccharide units (xylose and glucuronic acid) that binds to LG domains found in laminin and other proteins. It is the final result of Large glycosyltransferase activity.

**MuSK:** a receptor tyrosine kinase found in the muscle cell membrane that mediates neuromuscular junction formation in response to agrin binding.

**Neural dysplasia:** abnormal positioning of neuronal and glial cells in the nervous system as a result of disturbed development, either within or beyond their normal tissue borders, causing ectopic localization of mixed cellular populations (heterotopia).

**Neuromuscular junction (NMJ):** the synapse formed between the motor neuron and the muscle fiber, allowing motor impulses to induce muscle contraction.

**Node of Ranvier:** a section of neural axon that lacks a myelin sheath to allow ion permeability and action potential propagation. These gaps occur at regular intervals along the axon of all myelinated neurons.

**Pia mater:** the innermost layer of meningeal membranes lining the brain. It is composed of a heterogeneous population of cells including fibroblasts that secrete laminin and other matrix proteins to form the pial basement membrane at the surface of the brain.

**Pyridostigmine:** an acetylcholinesterase inhibitor used to enhance nerve-muscle signaling and reduce muscle weakness.

**Reelin:** a signaling molecule secreted during development by Cajal–Retzius cells in the hippocampus and neocortex. Reelin is critical for proper neuronal migration and function in both the developing and adult brain.

**Fig. 1.**
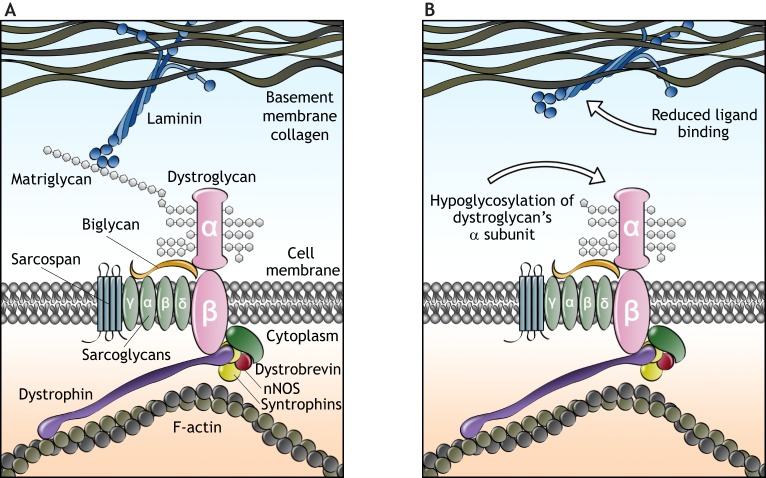
**Molecular pathogenesis of α-dystroglycanopathies.** (A) Schematic of dystroglycan protein interactions based on biochemical and functional evidence. A truncated form of αDG is most likely on display at the membrane surface, as the N-terminal region is cleaved during Golgi processing (Kanagawa et al., 2004). Matriglycan chains on the central mucin domain of αDG bind directly to laminin in the overlying basement membrane, while βDG is linked to the intracellular actin cytoskeleton through dystrophin. (B) Mutations in glycosyltransferase genes responsible for matriglycan construction cause a hypoglycosylation of αDG, resulting in loss of αDG-laminin binding and disruption of cell-matrix interaction. nNOS, neuronal nitric oxide synthase.

Aside from binding endogenous extracellular ligands, αDG is a receptor for *Mycobacterium leprae* – the organism responsible for leprosy – and for the Lassa hemorrhagic fever virus and the lymphocytic choriomeningitis virus (Cao et al., 1998; Rambukkana et al., 1998). The interaction between αDG and many of its extracellular binding partners is mediated by glycosylation (Box 2). Loss-of-function mutations in certain glycosyltransferase enzymes, discussed in more detail below, cause αDG hypoglycosylation (Box 2), which diminishes the binding affinity between αDG and its extracellular ligands (Fig. 1A,B).

In humans, the hypoglycosylation of αDG is characterized by progressive muscular dystrophy frequently associated with brain malformation and intellectual disability – a spectrum of recessive genetic disorders referred to as α-dystroglycanopathies. Researchers frequently use animal models of α-dystroglycanopathies to study the dysfunction of αDG in the brain, eye and peripheral nerve, because these are the least accessible components of the human pathology. Here, we summarize findings from these animal models and derive key inferences regarding the functions of αDG in the nervous system, with particular focus on their relevance to the human disorders.

## Molecular pathogenesis of α-dystroglycanopathies

αDG is so heavily glycosylated that carbohydrates constitute over half of the glycoprotein's mass. Of these carbohydrate structures, matriglycan (Box 2) is directly responsible for the binding of αDG to its ECM ligands. It is comprised of a massive repeating disaccharide (-3Xylα1-3GlcAβ1-) that tightly binds LG domains (Box 2) in the laminin, agrin (Box 2), perlecan, slit, neurexin and pikachurin extracellular proteins (Briggs et al., 2016). Matriglycans are synthesized by the enzyme Large and are bound to αDG through a tandem ribitol phosphate and a core O-linked trisaccharide (GalNAcβ1-3GlcNAcβ1-4Man-) (Kanagawa et al., 2016). The maximum quantity and repeat length of matriglycan molecules on αDG is not known.

**Fig. 2.**
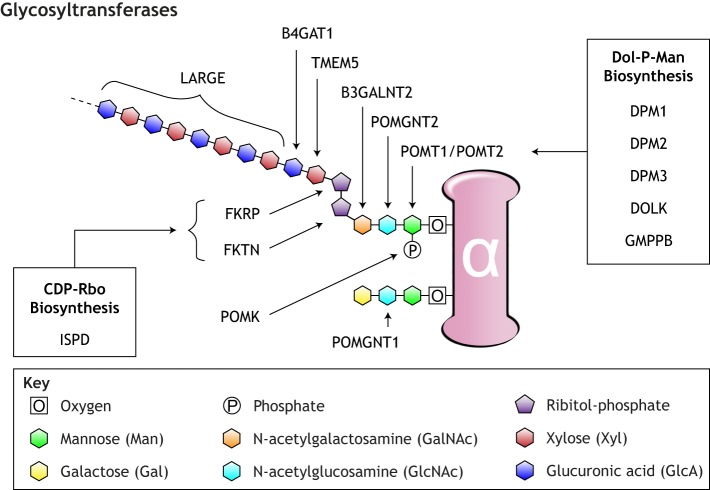
**Glycosylation of α-dystroglycan.** A simplified representation of the post-translational modifications on αDG, with arrows indicating each enzyme's respective glycan additions. POMT1/POMT2 catalyze the first step of glycosylation by adding an O-linked mannose to αDG in a process called O-mannosylation. The LARGE glycosyltransferase catalyzes the final step for the installation of the matriglycan, a repeating disaccharide of variable length that directly binds extracellular laminin. Other enzymes have been shown to be indirectly involved in αDG glycosylation through the synthesis of cytidine diphosphate ribitol (CDP-Rbo) and dolichol phosphate mannose (Dol-P-Man). Pathogenic mutations in each enzyme listed here are linked to the wide clinical spectrum of the α-dystroglycanopathies. For a detailed review, see Kanagawa and Toda, 2017.

To date, 16 genes have been identified as definitive contributors to the construction of functional matriglycans on αDG (Table 1, Fig. 2). A 17th gene, *POMGNT1*, is not directly involved in matriglycan synthesis but is thought to regulate its installation on αDG (Yoshida-Moriguchi et al., 2010). Loss of function in any of these genes compromises matriglycan structure and impairs αDG ligand-binding affinity, leading to a clinical condition called secondary α-dystroglycanopathy. In such cases, αDG maintains normal tissue expression and localization, but is hypoglycosylated (Box 2).

**Table 1.**
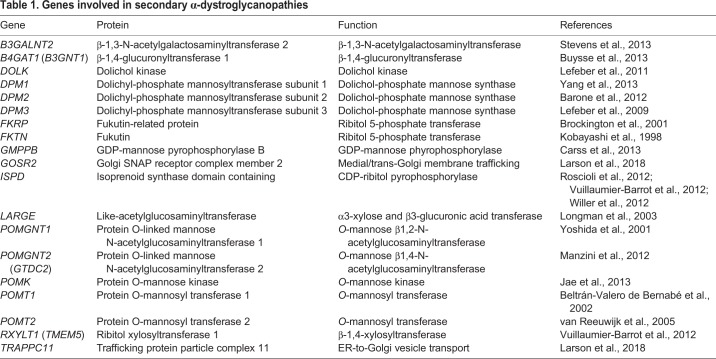
**Genes involved in secondary α-dystroglycanopathies**

Rare mutations in *DAG1* (Box 2), the gene encoding αDG and βDG, have also been reported to interfere with the process of matriglycan addition, either through inhibiting the extension of matriglycan (Hara et al., 2011) or by perturbing the maturation and trafficking of αDG (Geis et al., 2013; Signorino et al., 2018). Clinical conditions arising from mutations in DAG1 itself are referred to as primary α-dystroglycanopathies. Frameshift mutations in *DAG1* can completely abrogate αDG and βDG, resulting in particularly severe clinical manifestations (Riemersma et al., 2015). Often, but not always, the degree of αDG hypoglycosylation correlates with disease severity (Jimenez-Mallebrera et al., 2009; Alhamidi et al., 2017).

New genes continue to be implicated in α-dystroglycanopathies and further expand our view of αDG biosynthesis. Mutations in the Golgi membrane trafficking proteins TRAPPC11 and GOSR2 (Table 1) were recently reported to cause hypoglycosylation of αDG linked to muscular dystrophy, epilepsy and brain abnormalities (Larson et al., 2018). Given the more general nature of an ER-to-Golgi transport defect, it is likely that hypoglycosylated αDG is not the only disease mechanism for these two genes. Because many patients still lack a molecular diagnosis (Godfrey et al., 2007; Mercuri et al., 2009; Graziano et al., 2015), there are probably additional genes involved in α-dystroglycanopathies waiting to be identified.

## Neurological phenotypes in α-dystroglycanopathies

α-Dystroglycanopathies encompass a wide spectrum of disease severities that include disrupted nervous system development and progressive muscular dystrophy (see Box 3 for details on the muscular phenotypes of α-dystroglycanopathies). The most severely affected patients present with profound brain malformation and ultimately do not survive infancy – a phenotype referred to as Walker–Warburg syndrome (Cormand et al., 2001). Affected individuals can display profound cognitive deficits, hydrocephaly (Box 2), and brain and retinal dysplasia, some of which may be observed by ultrasound during gestation (Vohra et al., 1993; Trkova et al., 2015). Other syndromes recognized in the spectrum of α-dystroglycanopathy include (in order of decreasing severity) muscle-eye-brain (MEB) disease, Fukuyama-type congenital muscular dystrophy, and several forms of congenital and limb-girdle muscular dystrophies (reviewed by Godfrey et al., 2011).

**Box 3. Muscular phenotypes of α-dystroglycanopathies**

Muscular dystrophy is a central feature in α-dystroglycanopathies. In humans, muscular dystrophies are a large group of genetically mediated disorders of muscle, histologically characterized by muscle degeneration, attempted regeneration, and subsequent fibrosis and fatty replacement. Clinically, muscular dystrophies are extremely diverse but have progressive muscle weakness as a common feature. If the onset is pre-natal or around birth, they are referred to as congenital muscular dystrophies. Patients with severe α-dystroglycanopathy die in the first years of life, but mild forms can still be fatal due to intractable epilepsy or respiratory failure (Messina et al., 2009; Di Rosa et al., 2011; Pane et al., 2012).

Mice with global deletion of matriglycan-forming glycosyltransferases develop hallmarks of α-dystroglycanopathies, including muscular dystrophy and brain malformation. In addition to moderate cortical dysplasia, the *Large*^myd^ mouse shows an adult-onset progressive muscle wasting phenotype accompanied by stiffening limbs (Kelly et al., 1994). In contrast, *Fkrp* mutant mice (L276I/P448L and L276I/E310del) show a milder form of muscular dystrophy with no overt brain abnormalities (Blaeser et al., 2013). Interestingly, Cre-mediated deletion of *Dag1* in mature skeletal muscle causes only a mild dystrophy, as regeneration is aided by αDG-expressing muscle progenitor cells (Cohn et al., 2002). Thus, in addition to its various roles in the nervous system discussed in this Review, αDG critically maintains differentiated and regenerating muscle fibers.

**Fig. 3.**
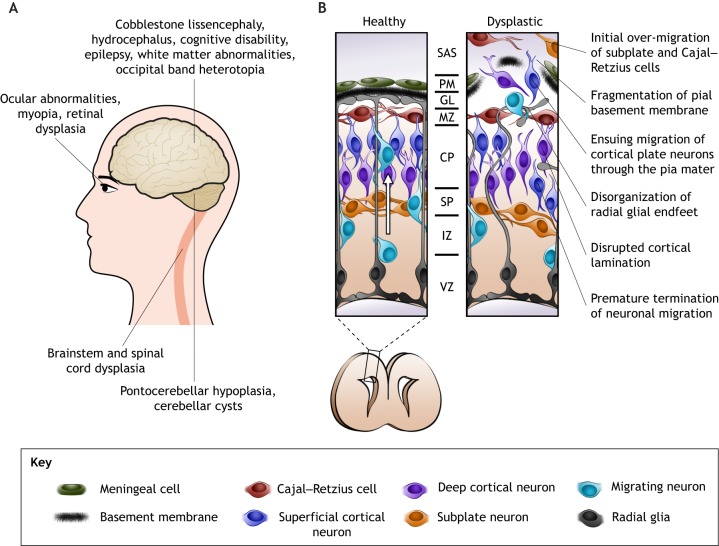
**Neural phenotypes in α-dystroglycanopathies.** (A) Diagram of the nervous system regions primarily affected in α-dystroglycanopathies. Gross malformations are commonly reported in the brain and eyes and can include displaced neurons and glia (heterotopia), and abnormally small pons and cerebellum (pontocerebellar hypoplasia). These structural phenotypes are often accompanied by functional deficits in cognition and vision (myopia) (Santavuori et al., 1989; Pihko et al., 1995; Cormand et al., 2001; von Renesse et al., 2014). (B) Healthy brain development involves radial migration of newborn neurons (white arrow) into laminae of the cortical plate. Radial glia anchored to the pial basement membrane act as a guiding scaffold. Cortical dysplasia in α-dystroglycanopathy models is characterized by discontinuity of the pial basement membrane, disorganization of radial glial endfeet, abnormal migration of cells into the subarachnoid space and disrupted cortical lamination. CP, cortical plate; GL, glia limitans; IZ, intermediate zone; MZ, marginal zone; PM, pia mater; SAS, subarachnoid space; SP, subplate; VZ, ventricular zone.

A common feature of severe α-dystroglycanopathies is cobblestone lissencephaly (Box 2) and an array of neurological defects (Fig. 3A). Light and electron microscopy of postmortem tissue establish a general theme of aberrant cell migration underlying such nervous system dysplasia (Nakano et al., 1996). Examination of the cerebral cortex, cerebellum, retina, brainstem and spinal cord shows gross morphological abnormalities due to displacement of neurons both within the tissue and beyond its normal borders (Yamamoto et al., 1997, 2008). In some cases, there is evidence of atrophy in the spinal cord and retina (Meilleur et al., 2014), sometimes accompanied by progressive dysfunction of ocular physiology (Santavuori et al., 1989; Pihko et al., 1995; Cormand et al., 2001; von Renesse et al., 2014).

Over two decades of accumulated research triangulates αDG dysfunction as a primary causative mechanism for this class of muscular dystrophies with central nervous system involvement. In mice, disruption of the genes involved in the synthesis of matriglycans on αDG, or in *Dag1* itself, results in animals that recapitulate the human condition in many respects (Michele et al., 2002; Moore et al., 2002). Despite our knowledge regarding the genetic etiology of α-dystroglycanopathies, the pathogenic events leading to such a heterogeneous spectrum of disease remain unclear.

## Considerations for the use of α-dystroglycanopathy models

An increasingly rich assortment of model systems is currently available for investigating α-dystroglycanopathies. Interpreting the literature necessitates considering the strengths and weaknesses of each model, and selecting the best model requires factoring these caveats into the goals of future studies.

Traditionally, mice have been the preferred animal model for α-dystroglycanopathies. There are at least 41 distinct genetic mouse models: 15 that directly mutate or delete *Dag1* itself (Table 2), and 26 that model the hypoglycosylation of αDG through mutation or deletion of glycosyltransferase genes (Tables 3 and 4). Global deletion of *Dag1* or the glycosyltransferases *Pomt1*, *Pomt2*, *Fktn* or *Fkrp* is embryonic lethal in mice due to disruption of Reichert's membrane, a murine basement membrane barrier between embryonic and placental tissue (Williamson et al., 1997; Willer et al., 2004; Kurahashi et al., 2005; Chan et al., 2010; Hu et al., 2011a,b). Defects in Reichert's membrane also occur upon knockout of glycosyltransferase enzymes involved in matriglycan synthesis (Willer et al., 2004; Kurahashi et al., 2005; Chan et al., 2010; Hu et al., 2011a). This membrane is not present in human embryos, and thus these models are only practical for assessing the role of αDG in early murine development.

**Table 2.**
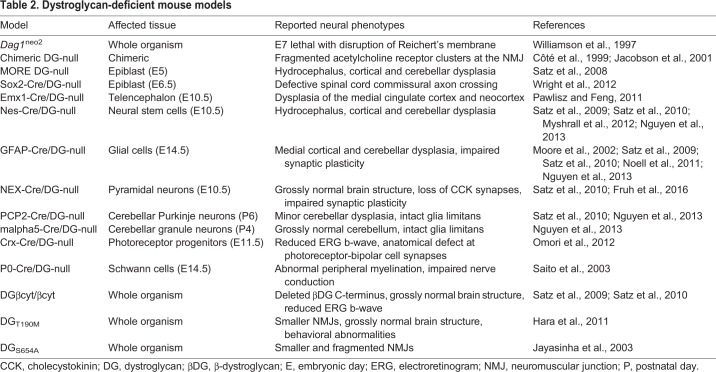
**Dystroglycan-deficient mouse models**

**Table 3.**
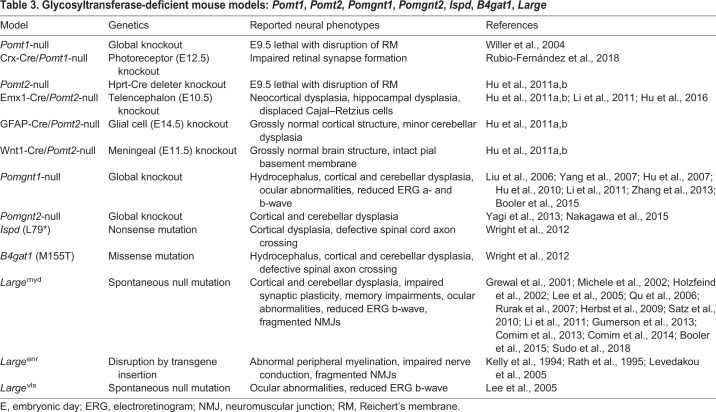
**Glycosyltransferase-deficient mouse models: *Pomt1*, *Pomt2, Pomgnt1*, *Pomgnt2*, *Ispd*, *B4gat1*, *Large***

**Table 4.**
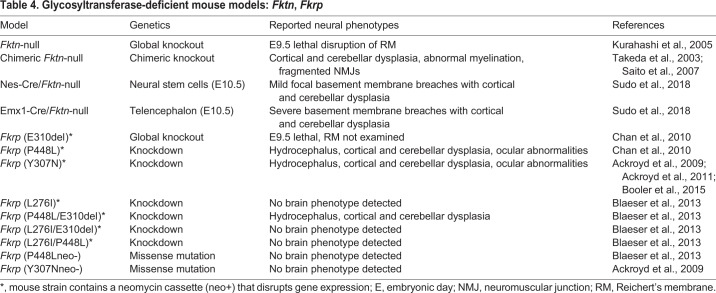
**Glycosyltransferase-deficient mouse models: *Fktn*, *Fkrp***

Many Cre-driver mouse lines (Box 2) are used for tissue-specific deletion of *Dag1*, allowing embryonic survival and therefore the study of phenotypes in older animals. Particularly useful have been the GFAP-Cre, NEX (also known as Neurod6)-Cre and Nes (also known as nestin)-Cre mice, which drive recombination in glia, neurons and neural stem cells, respectively. Crossing these lines with floxed *Dag1* mice is a common strategy for investigating dystroglycan's functional contributions in distinct cell types (Satz et al., 2010). Neuron subtype-selective approaches are also possible – for example, Pcp2-Cre mice exhibit recombination primarily in cerebellar Purkinje neurons (Nguyen et al., 2013).

A recent comparison of α-dystroglycanopathy mouse models demonstrated significant phenotypic differences depending on the Cre-driving promoter. Gene knockout driven by Nes-Cre showed a considerably milder effect compared with that driven by Emx1-Cre (Sudo et al., 2018). Neural stem cells of the cerebral cortex express both Nes and Emx1, but Nes-Cre-driven knockout was inefficient in early stages of development (Liang et al., 2012). Because the timing of αDG loss of function has important phenotypic consequences, Emx1-Cre mice might be a preferred system for investigating the functional contribution of αDG during fetal brain development.

Knockdown of glycosyltransferase genes, rather than complete ablation, is another common approach to generate viable postnatal mouse models of α-dystroglycanopathies. For example, various levels of knockdown in *Fkrp* – a glycosyltransferase critical for matriglycan synthesis – mimic the broad clinical variability seen in α-dystroglycanopathies (Chan et al., 2010; Blaeser et al., 2013). A comparison between *Fkrp*, *Pomgnt1* and *Large* (also known as *Large1*; Box 2) mutant mouse strains has elucidated gene-specific phenotypes and exemplifies the utility of these diverse models (Booler et al., 2015).

In addition to mouse models, zebrafish are commonly used owing to their short life cycle and feasibility of targeted gene knockdown with morpholinos (Table 5). Although comparisons between zebrafish and human phenotypes are limited, it is a useful strategy for rapidly confirming the functional significance of newly identified genes in α-dystroglycanopathies. Other non-mammalian models include chick, *Xenopus*, *Drosophila* and *Caenorhabditis elegans*, as these systems are amenable for high-throughput genetic screens or allow greater access for experimental manipulation. This abundance of animal models allows for a flexible approach to studying the genetic and phenotypic spectrum of α-dystroglycanopathies.

**Table 5.**
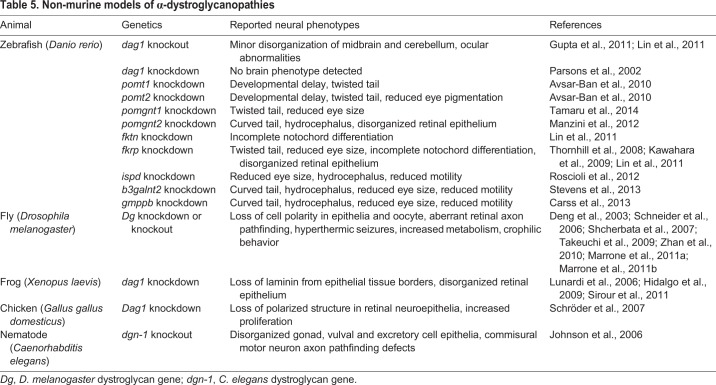
**Non-murine models of α-dystroglycanopathies**

## α-Dystroglycan and the structural integrity of the nervous system

### Matrix organization

Patients with α-dystroglycanopathy show disruption of basement membranes in both muscle and brain (Saito et al., 1999; Vajsar et al., 2000; Goddeeris et al., 2013). Basement membrane synthesis is believed to begin with soluble extracellular laminin binding to cell membrane galactosyl-sulfatide glycolipids (Li et al., 2005). At high local concentrations, laminins polymerize to form a network at the cell surface, recruiting additional basement membrane proteins and mobilizing integrin and αDG to strengthen the nascent matrix. Thus, αDG is not required for initial basement membrane formation, but likely participates in its maintenance.

As in humans, loss of functional αDG in experimental animal models results in gross malformation to the nervous system, including lissencephaly and hydrocephaly (Satz et al., 2010). Such phenotypes are often accompanied by microscopic ruptures in basement membranes of the neocortex, hippocampus, cerebellum, retina and spinal cord, with abnormal migration of neurons through these breaches (Ackroyd et al., 2009; Li et al., 2011; Wright et al., 2012; Nguyen et al., 2013).

Because *Dag1*-null mouse embryos do not survive gastrulation, other methods have been sought to probe the role of αDG in basement membrane integrity. Mouse embryonic stem cells can be cultured in suspension to form embryoid bodies (Box 2). An initial report observed complete absence of basement membrane in embryoid bodies derived from *Dag1*-null embryonic stem cells (Henry and Campbell, 1998), but later studies show that such *Dag1*-null embryoid bodies can, in fact, form a basement membrane (Li et al., 2002).

Basement membranes produced in *Dag1*-null embryoid bodies can develop an abnormally thick morphology, suggesting reduced compaction or increased matrix secretion (Li et al., 2002). The epiblast-like cells of embryoid bodies express both αDG and integrin, two major classes of cell surface laminin receptors. In the absence of either receptor, the other can compensate to assemble a basement membrane if high concentrations of laminin proteins are present. However, knockout of both receptors prevents basement membrane formation entirely (Li et al., 2017). Although αDG is not required for the initial polymerization of laminin (Li et al., 2005), it may reinforce and compact the basement membrane through collateral linkages to laminin, agrin and perlecan (Goddeeris et al., 2013).

When *Dag1* knockout is restricted to the epiblast of the developing mouse, the embryo successfully progresses through gastrulation but develops severe malformation of the nervous system (Satz et al., 2008). Interestingly, selective deletion of *Dag1* from various neuron subtypes in NEX-, PCP2- and malpha6 (also known as Tuba1c)-Cre mice, or from the meninges in Wnt1-Cre mice, does not cause nervous system malformation (Satz et al., 2009, 2010; Hu et al., 2011a; Nguyen et al., 2013). Likewise, removing the cytoplasmic C-terminus of βDG has no apparent effect (Satz et al., 2010).

However, αDG is highly expressed in glial cells contacting the basement membranes that ensheath the brain, retina and neural vasculature (Zaccaria et al., 2001). Knockout of glial *Dag1* in GFAP-Cre mice leads to fragmentation of basement membranes overlying the cerebral cortex and cerebellum (Moore et al., 2002; Satz et al., 2010; Nguyen et al., 2013). Various mouse models expressing a hypoglycosylated αDG also show similarly disrupted basement membranes (Tables 3 and 4) (Michele et al., 2002; Lee et al., 2005; Satz et al., 2010; Takahashi et al., 2011). Together, these animal models suggest that αDG has an important receptor function in glial cells involving the integrity of brain ECM structure.

### Cortical histogenesis

During mammalian brain development, neuroepithelial cells extend from the ventricular zone to contact the pia mater (Box 2) at the brain's surface (McLone, 1980). These neuroepithelial cells, which later differentiate into radial glia, form a layer of endfoot processes, called the glia limitans, in close apposition to the pia mater (Fig. 3B). Meningeal fibroblasts of the pia mater deposit ECM components that are sandwiched to form a basement membrane between the pia mater and underlying glial endfeet (Sievers et al., 1994). Radial glia rely on the basement membrane to maintain their morphology and localization. In turn, they remodel and strengthen its framework through receptor-matrix interactions (Sievers et al., 1986). *In vitro* cultures of meningeal cells and glia spontaneously form basement membrane structures at the interface between the two cell types, demonstrating that both the meninges and glia are necessary for the formation of the pial basement membrane (Abnet et al., 1991; Struckhoff, 1995). Importantly, the pial basement membrane and its associated glial endfeet act as a scaffold for neuronal migration and establishment of the brain's cellular architecture (Halfter et al., 2002).

Ruptures in the pial basement membrane are characteristic of severe forms of α-dystroglycanopathy (Fig. 3B). Such ruptures correlate with disorganized glial endfeet and abnormal neuronal migration (Hu et al., 2007; Yang et al., 2007). A common feature is the accumulation of neurons beyond the cortical boundary, presumably due to over-migration through basement membrane breaches (Booler et al., 2015). The underlying cortical plate is also disrupted by irregular neuronal orientation and premature termination of migration, interrupting the two major modes of radial neuronal migration: radial glia-guided and radial glia-independent migration (Nakagawa et al., 2015). This is speculatively due to the apparent disorganization of glial processes and fragmented basement membrane, respectively. Similarly, both the hippocampus and the cerebellum experience neuronal under-migration, resulting in abnormal morphology (Liu et al., 2006; Nguyen et al., 2013). Additional neuronal migration defects can be found in the retina, hindbrain and spinal cord across mouse, zebrafish and *Drosophila* models (Table 5) (Lunardi et al., 2006; Qu et al., 2006; Shcherbata et al., 2007; Thornhill et al., 2008; Kawahara et al., 2009; Gupta et al., 2011; Marrone et al., 2011a,b; Wright et al., 2012).

A subset of α-dystroglycanopathy patients develop both pachygyria and polymicrogyria – abnormally thick and thin convolutions of the cerebral cortex, respectively – specifically in the frontal and parietal lobes (Aida et al., 1996; Meilleur et al., 2014; Yoshioka et al., 2017). Occipital subcortical heterotopia, cerebellar cysts and other defects across many brain regions also occur (Clement et al., 2008). In contrast, brain malformations in *Pomgnt1*-null and *Fkrp*(Y307N) mice follow a lateromedial and rostrocaudal gradient of severity. *Large*^myd^ mice show a more consistent abundance of lesions across the midbrain and cortex (Booler et al., 2015). There are no reports of a lesion gradient in α-dystroglycanopathy patients that match that of *Pomgnt1*-null and *Fkrp*(Y307N) mice. The underlying mechanisms behind such diversity of neural dysplasia (Box 2) between patients and mice are unknown. Remarkably, despite widespread disorganization to the brain's usually laminar architecture, many neurons still differentially express layer-specific markers and project to appropriate brain regions in mouse models (Myshrall et al., 2012). This indicates that some axon pathfinding mechanisms are independent of cortical lamination and may help explain the rather mild cognitive dysfunction in some patients with otherwise extensive brain malformation (Godfrey et al., 2007; Meilleur et al., 2014).

Detailed examination of α-dystroglycanopathy model embryos has highlighted pathologic features that mark the onset of abnormal brain development. In *Pomgnt2*-null and *Fkrp*(Y307N) mice, ectopic movement of Cajal–Retzius cells and subplate neurons through the pial basement membrane occurs as early as embryonic day 10.5 (Nakagawa et al., 2015; Booler et al., 2016). This is followed by cortical plate neurons streaming through the pial ruptures, accumulating beyond the brain's surface in the subarachnoid space. Cajal–Retzius cells orchestrate cortical lamination by secreting reelin (Box 2) (Ogawa et al., 1995). Thus, this early mislocalization of Cajal–Retzius cells might influence later positioning of cortical plate neurons. However, biochemical analyses indicate that there is no perturbation to global reelin signaling in *Pomgnt2*-null and *Fkrp*(Y307N) mouse brains (Nakagawa et al., 2015; Booler et al., 2016).

Meningeal cells of the pia mater and their associated pial basement membrane provide signaling cues and a physical substrate for the migration of Cajal–Retzius cells and other neurons (Borrell and Marín, 2006; Sekine et al., 2012). In α-dystroglycanopathies, the pial basement membrane is believed to form abnormally, potentially rendering it prone to discontinuity and creating a permissive environment for migrating cells to cross its boundary (Hu et al., 2007). Indeed, atomic force microscopy reveals that the retina's inner limiting basement membrane in *Pomgnt1*-null mice is roughly half as stiff and half as thick as that in wild-type mice (Hu et al., 2010; Zhang et al., 2013). Further, *Pomgnt1*-null neural stem cells demonstrate up to 60% slower accumulation of surface laminin compared with wild type, consistent with reduced ECM assembly by αDG-deficient cells (Zhang et al., 2013).

Interestingly, mutations in ECM receptor genes other than *Dag1* can similarly cause basement membrane breaches. Both GPR56 and integrin-β1 – cell surface receptors of collagen III and laminins, respectively – are expressed on radial glial endfeet abutting the pial basement membrane. Knockout of these receptors causes mislocalization of glial endfeet and aberrant neuronal migration at fragmented regions of the pia mater, ultimately leading to a cobblestone lissencephaly-like cortical dysplasia (Graus-Porta et al., 2001; Li et al., 2008).

Directly perturbing the ECM can also result in pathologic conditions reminiscent of α-dystroglycanopathy. Patients with mutations in laminin subunits α2 and β1 often develop localized cobblestone lissencephaly in the occipital lobe (Geranmayeh et al., 2010; Radmanesh et al., 2013). In animal models, neuronal migration defects are elicited through the perturbation of laminin-α1, laminin-β2, laminin-γ1, laminin-γ3, collagen III α1, collagen IV α1 or collagen IV α2 (Halfter et al., 2002; Pöschl et al., 2004; Luo et al., 2011; Ichikawa-Tomikawa et al., 2012; Radner et al., 2013).

Because disruption of either ECM receptors or the pia mater ECM itself results in comparable pathology, receptors such as αDG are likely to be involved in physically strengthening the pial basement membrane. However, given that αDG is expressed in a wide array of brain cells beyond radial glia, as discussed in detail below, additional factors are surely necessary to account for all disease-related phenomena seen in patients. This is particularly true for patients with mutations affecting the earliest steps of O-mannosylation, which may affect other glycosylated proteins in addition to αDG (Lommel et al., 2013; Vester-Christensen et al., 2013). However, recent work indicates that cadherins and plexins, two major cell surface receptor families, are not modified by the same O-mannosylation enzymes as αDG (Larsen et al., 2017). Therefore, we consider that the impairment of pial basement membrane integrity from specific loss of αDG-matrix interaction is a likely pathogenic mechanism for the neuronal migration defects in α-dystroglycanopathies.

## Focal organization of the nervous system

### Cell polarity

Although gross malformation to the nervous system is common in α-dystroglycanopathies, increasing evidence suggests perturbation at the subcellular level. Abnormalities in cell polarity, channel distribution and cellular signaling could influence the disease phenotype and be intimately tied to the development of brain dysplasia. As a bridge between extracellular and intracellular elements, αDG and βDG coordinate cytosolic proteins with external cues. Thus, in addition to ECM organization, binding between αDG and its ligands may localize subcellular specializations through interaction with βDG (Moore and Winder, 2010). There is also accumulating evidence that βDG can translocate to the nucleus, regulating nuclear envelope structure and gene expression (Martínez-Vieyra et al., 2013; Mathew et al., 2013; Gracida-Jiménez et al., 2017).

In the embryonic chick retina, DAG1 knockdown induces neuroepithelial stem cell detachment from the inner limiting basement membrane, with a concomitant loss of elongated cell morphology and stalling of apicobasal interkinetic nuclear migration (Schröder et al., 2007). Displacement of molecular markers of epithelial polarity is commonly observed in Dg-null *Drosophila* (Deng et al., 2003; Schneider et al., 2006; Shcherbata et al., 2007). Further, binding between laminin and αDG in mouse embryoid bodies is sufficient to induce polarized morphology of epiblast-like cells and regulate apicobasal orientation (Li et al., 2017). Although the molecular mechanisms have not been precisely defined, there are accumulating data that asymmetrically distributed αDG-laminin interactions can determine cell polarization at basement membrane contact points, perhaps involving cytoplasmic microtubules, CLASP or PAR-1 proteins (Masuda-Hirata et al., 2009; Nakaya et al., 2013).

In the brain and retina, αDG colocalizes with aquaporin-4 water channels and Kir4.1 potassium channels at perivascular glial endfoot processes (Guadagno and Moukhles, 2004). Co-immunoprecipitation experiments showed that both aquaporin-4 and Kir4.1 associate with members of the dystrophin-glycoprotein complex, including βDG (Fort et al., 2008). Binding between αDG and laminin and agrin apparently anchors aquaporin-4 and Kir4.1 within specialized domains at glial endfeet, possibly regulating ion and water homeostasis in the brain (Noel et al., 2005). Animal models of α-dystroglycanopathies accordingly show a deficit of aquaporin-4 and Kir4.1 channels in perivascular glial endfeet (Michele et al., 2002; Rurak et al., 2007; Noell et al., 2011). Notably, deletion of the cytoplasmic C-terminus of βDG is sufficient to reduce Kir4.1 channel clustering in retinal radial glia and produce a reduced electroretinogram b-wave (Box 2), indicating abnormal visual signal transmission in the retina (Satz et al., 2009). Downregulation of βDG and aquaporin-4 in glial endfeet also occurs after epileptic activity (Gondo et al., 2014). This channel clustering defect may broadly perturb interstitial brain water homeostasis and be linked to white matter abnormalities in α-dystroglycanopathy patients (Aida et al., 1996; Cormand et al., 2001; Larson et al., 2018).

### Myelination

In addition to the endfeet of radial and perivascular glia, αDG is expressed in myelinating glia at various stages of maturity (Yamada et al., 1994; Colognato et al., 2007). White matter alterations are frequently seen on brain magnetic resonance imaging (MRI) scans of α-dystroglycanopathy patients, particularly in those with intellectual deficits (Bönnemann et al., 2014). As discussed in the preceding section, some of these signal alterations may be related to an abnormal water content of the white matter owing to aberrant channel function in radial and perivascular glial endfeet. However, further observations also suggest a pathology of myelinated fiber tracts in α-dystroglycanopathies.

In the peripheral nervous system, αDG localizes to the outer aspect of Schwann cell membranes in contact with the overlying basement membrane and is upregulated during the myelination of regenerating nerves (Masaki et al., 2000). Proper folding of the myelin sheath relies on the collective laminin binding activity of αDG and integrin-α6β4 (Nodari et al., 2008). Additionally, αDG interactions with laminin-211 may guide Schwann cell microvilli to axoglial junctions and thus indirectly mediate sodium channel clustering at the node of Ranvier (Box 2) (Saito et al., 2003). Loss of αDG-laminin binding in mice impairs nerve regeneration, myelination and signal conduction with associated behavioral deficits. In multiple mouse models of α-dystroglycanopathies, researchers reported an axon-sorting defect in the sciatic nerve characterized by amyelinated axon bundles, some of which were large enough to expect myelination (Kelly et al., 1994; Rath et al., 1995; Levedakou et al., 2005; Saito et al., 2007). This type of pathology might not be a major feature of the human disease, as it has not yet been documented in patients.

In the central nervous system, αDG is expressed on developing oligodendrocytes, where it associates with laminin and IGF-1 signaling proteins, suggesting a role in oligodendrocyte morphological maturation and differentiation (Galvin et al., 2010). Knockout of *Dag1* in neural stem cells compromises the ECM structure of the subventricular zone, a major region of origin for oligodendrocytes, and impedes the maturation of ependymal cells and oligodendrocytes (McClenahan et al., 2016). Further, *in vitro* disruption of αDG-laminin binding leads to decreased filopodia formation and myelination by oligodendrocytes (Colognato et al., 2007; Eyermann et al., 2012). Collectively, these data place αDG as a physical link between the myelinating cell membrane and the ECM, perhaps acting to regulate its proliferation and differentiation or even to stabilize and guide the process of myelination itself.

### Organization of the neuromuscular junction

αDG is expressed at peripheral nervous system synapses, where it serves apparent scaffolding functions distinct from that of glial-expressed αDG (Bewick et al., 1993; Zaccaria et al., 2001). A subset of α-dystroglycanopathy patients with mutations in *GMPPB* (Table 1) exhibit striking decreases in muscle action potentials during repeated nerve stimulation. Pyridostigmine (Box 2) treatment reportedly improves motor function in these patients, suggesting an abnormality of neuromuscular transmission at the postsynaptic neuromuscular junction (NMJ; Box 2) (Belaya et al., 2015; Rodríguez Cruz et al., 2016). However, other α-dystroglycanopathy patients do not show abnormalities, and the reason for these differences remains unknown.

αDG resides at the NMJ postsynaptic membrane and was the first identified receptor of agrin, a master organizer of the NMJ that stimulates acetylcholine receptor (AChR) clustering through its interaction with postsynaptic receptors (Bowe et al., 1994; Campanelli et al., 1994; Gee et al., 1994; Sugiyama et al., 1994). Knockout of *Dag1* in mice does not prevent NMJ formation – instead, NMJs are smaller with fragmented AChR clusters (Côté et al., 1999; Jacobson et al., 2001). This is likely because MuSK (Box 2) is the primary agrin receptor required for NMJ formation, while αDG and βDG appear to be involved in a MuSK-independent mechanism to stabilize AChR clusters (DeChiara et al., 1996; Glass et al., 1996; Cartaud et al., 1998; Jacobson et al., 1998).

In addition to agrin, αDG binds extracellular perlecan at the NMJ (Peng et al., 1998). Perlecan, in turn, binds and stabilizes acetylcholinesterase (AChE) in the synaptic basement membrane (Peng et al., 1999). Because perlecan-null mice show complete absence of AChE at the NMJ, it is possible that neuromuscular transmission in α-dystroglycanopathies may also be impaired by a disruption of an AChE-perlecan-αDG complex (Arikawa-Hirasawa et al., 2002). Chimeric *Dag1*-null mice exhibit reduced levels of AChE at the NMJ (Côté et al., 1999; Jacobson et al., 2001); however, a functional consequence of AChE disruption has not yet been shown in the α-dystroglycanopathies or its models.

### Organization of central synapses

In apparent similarity to the NMJ, αDG is also expressed at postsynaptic specializations of the central nervous system (Zaccaria et al., 2001). Cognitive impairment and epilepsy are features in many α-dystroglycanopathies (Messina et al., 2009; Di Rosa et al., 2011; Astrea et al., 2018; Larson et al., 2018). For some patients, MRI shows grossly normal brain morphology despite significant cognitive deficits (Godfrey et al., 2007; Clement et al., 2008; Jimenez-Mallebrera et al., 2009). This observation suggests a neuronal dysfunction below the detection limit of current brain imaging tools, such as central synaptic dysfunction, for example.

The expression of αDG in central neurons is restricted to a subset of postsynaptic sites in pyramidal cells of the cerebral cortex and hippocampus as well as cerebellar Purkinje cells (Zaccaria et al., 2001). Immunolabeling of murine hippocampal cultures reveals a selective association between αDG and postsynaptic proteins of γ-aminobutyric acid (GABA) inhibitory synapses: dystrophin, GAD, gephyrin and GABA_A_ receptor subunits α1, β2/3 and γ2 (Brünig et al., 2002; Lévi et al., 2002; Pribiag et al., 2014). Because αDG is an extracellular membrane-associated protein, it is reasonable to speculate that it may act as part of a trans-synaptic protein complex, perhaps facilitating synapse formation or maintenance. Indeed, characterized ligands of αDG, agrin and the neurexin proteins, are expressed at presynaptic terminals in the brain where they may interact across the synapse with matriglycans on postsynaptic αDG (Ferreira, 1999; Sugita et al., 2001).

The NEX-Cre/DG-null mouse, in which *Dag1* is conditionally deleted from pyramidal neurons, shows virtually complete loss of cholecystokinin (CCK) GABAergic presynaptic terminals (Fruh et al., 2016). Despite this selective loss of presynaptic input, there is no change in the total number of GABAergic presynaptic terminals, suggesting a compensatory effect. This is consistent with the *in vitro* observation that *Dag1* knockout does not affect overall GABAergic synapse numbers (Lévi et al., 2002). Knockout of *Dag1* after synapse formation in adult animals likewise causes selective loss of CCK terminals, but there is no such phenotype in the *Dag1* T190M knock-in mouse – a model of α-dystroglycanopathy expressing a neurexin-binding-deficient αDG (Fruh et al., 2016). Because this process appears independent from αDG-neurexin binding, αDG may interact with additional unidentified presynaptic receptors or indirectly control other trans-synaptic proteins to facilitate CCK GABAergic innervation.

In primary hippocampal cultures, a pharmacological increase in neuronal firing rate induces a compensatory boost in inhibitory synaptic strength, accompanied by increased clustering of αDG and GABA_A_Rs at the postsynaptic surface (Pribiag et al., 2014). Interestingly, this inhibitory upscaling is partly reproduced by the addition of agrin to the culture medium, and it is blocked by knockdown of *Dag1* or the glycosyltransferase Large, demonstrating that the receptor function of αDG is required for this form of homeostatic synaptic plasticity. The inhibitory synaptic defects identified in mouse and cellular models may explain the epilepsy seen in patients (Pribiag et al., 2014; Fruh et al., 2016). However, future work must delineate the various biochemical interactions of αDG and βDG and their potential roles in synapse formation versus synapse plasticity.

### Organization of retinal synapses

While αDG resides at the postsynaptic apparatus of some central synapses and the NMJ, it assumes a unique presynaptic position in the retina. Early work identified αDG and βDG, in association with dystrophin, within the outer plexiform layer, where presynaptic photoreceptor terminals contact postsynaptic bipolar and horizontal cells (Montanaro et al., 1995). Interestingly, both α-dystroglycanopathy patients and animal models present abnormal electroretinograms (ERGs; Box 2), indicating retinal dysfunction (Holzfeind et al., 2002; Takeda et al., 2003; Lee et al., 2005). Some patients, in particular those with the *POMGNT1* mutations prevalent in Finnish MEB patients, show overt evidence for retinal degeneration (Santavuori et al., 1989; Pihko et al., 1995).

The synaptic ligand of αDG in the retina, encoded by pikachurin (also known as *Egflam*), was discovered in a transcriptomic screen comparing retinas from wild-type and *Otx2*-null mice, a mouse strain lacking photoreceptor cells (Sato et al., 2008). Pikachurin localizes to the synaptic cleft of the specialized ribbon synapse between photoreceptor and bipolar cells. There, it binds the matriglycans on αDG to link presynaptic rod/cone photoreceptors to the postsynaptic bipolar cell dendrite, allowing for rapid communication across the first synapse in the visual system (Kanagawa et al., 2010; Hu et al., 2011b). A recent analysis demonstrates that pikachurin acts as a trans-synaptic bridge, physically joining presynaptic αDG to the postsynaptic orphan receptor GPR179 (Orlandi et al., 2018).

Pikachurin and αDG are mutually required for proper localization, because ablation of either causes a significant reduction to the other (Omori et al., 2012). Ultrastructurally, loss of the photoreceptor αDG-pikachurin complex leads to abnormal synapse formation between photoreceptors and bipolar cells (Sato et al., 2008; Omori et al., 2012; Rubio-Fernández et al., 2018). This uncoupling of the photoreceptor from the bipolar cell was confirmed by ERG in both mice and patients, where the a-wave is unimpaired but the b-wave exhibits a significantly attenuated amplitude (Santavuori et al., 1989; Pihko et al., 1995; Longman et al., 2003; Sato et al., 2008; Omori et al., 2012; Rubio-Fernández et al., 2018).

## Conclusions and perspective

Based on the wealth of data now available from various experimental animal models, we propose at least two broad, but distinct, categories of nervous system pathology in α-dystroglycanopathies: (1) a histological abnormality arising from dysfunction of glial αDG, largely responsible for the distinctive migration abnormalities during brain development and white matter changes; and (2) a synaptic defect from dysfunction of neuronal αDG. α-Dystroglycanopathy patients often present with intellectual deficits presumably caused by developmental brain malformation. However, an increasingly recognized subset of patients present with significant cognitive delays but normal or near-normal brain structure as determined by MRI (Godfrey et al., 2007; Clement et al., 2008; Jimenez-Mallebrera et al., 2009). We hypothesize that these individuals may harbor a selective defect at the synapse level that contributes to their cognitive dysfunction. This will be important to identify, as synaptic pathology could potentially be amenable to treatments that are targeted at improved αDG function (Box 4).

**Box 4. Therapeutic approaches for α-dystroglycanopathies**

Animal models are an important pre-clinical platform for α-dystroglycanopathy therapy development. Forced overexpression of wild-type *LARGE*, the glycosyltransferase directly responsible for matriglycan synthesis, has been shown to restore αDG glycosylation (Barresi et al., 2004). This approach significantly increases αDG-laminin binding and improves muscle function in the *Large*^myd^ mouse (Barresi et al., 2004; Hildyard et al., 2016). This therapeutic strategy may extend to multiple forms of α-dystroglycanopathy, as *LARGE* overexpression enhances αDG glycosylation in human cells and mouse models with mutations in *POMT1*, *POMGNT1*, *FKTN* and *FKRP* (Barresi et al., 2004; Yu et al., 2013; Vannoy et al., 2014). However, *LARGE* replacement therapy in *Fktn*- and *Fkrp*-null mice has also been shown to worsen muscle pathology (Whitmore et al., 2013; Saito et al., 2014). These conflicting results warrant further investigation into glycosylation-modulating therapies before moving to the clinic.

The post-translational addition of ribitol to αDG is a precursor step to matriglycan synthesis. Because this process is catalyzed by ISPD, FKTN and FKRP, α-dystroglycanopathy patients carrying mutations in these enzymes might benefit from ribitol treatment. Encouragingly, supplementation of ribitol in cell culture medium enhances αDG glycosylation in *ISPD* mutant cells (Gerin et al., 2016; Kanagawa et al., 2016), and dietary administration in *Fkrp*(P448L) mice diminishes skeletal muscle phenotypes (Cataldi et al., 2018).

Restoring αDG glycosylation could also ameliorate the intellectual deficits associated with α-dystroglycanopathies. Transgenic expression or gene delivery of glycosyltransferases in the brain restores wild-type levels of glycosylation to αDG and prevents abnormal cortical development in mouse models (Hildyard et al., 2016; Sudo et al., 2018). Further, postnatal AAV9-mediated gene delivery of wild-type *Dag1* improved cognitive function in the Emx1-Cre/*Pomt2*-null mouse, despite persistent cortical dysplasia (Hu et al., 2016). This promising result suggests that even patients with considerable brain malformation may experience behavioral improvement with therapy.

Few studies have comprehensively assessed cognitive ability in α-dystroglycanopathies, but some reports indicate impaired executive control, memory and visuospatial attention in patients with macroscopically normal brain structure or minimal brain malformation (Palmieri et al., 2011; Meilleur et al., 2014). The *Large*^myd^ and Emx1-Cre/DG-null mouse models of α-dystroglycanopathy similarly show a memory consolidation deficit (Comim et al., 2013, 2014; Hu et al., 2016). Attention and memory are correlated with gamma oscillations of 30-80 Hz in the cortex – a frequency of electrical activity synchronized partly by CCK interneurons (Engel et al., 2001; Tukker et al., 2007). Interestingly, as discussed earlier, the NEX-Cre/DG-null mouse displays a selective loss of CCK GABAergic synaptic inputs (Fruh et al., 2016). Considering these results, gamma oscillations could be studied in α-dystroglycanopathies, especially given that abnormal gamma oscillations have already been reported in other brain disorders, including autism, epilepsy and schizophrenia (Uhlhaas and Singer, 2006). The NEX-Cre/DG-null line is particularly suited to these investigations, as it avoids the confounding factor of brain malformation by selective knockout of neuronal *Dag1*.

Much is still unknown about the role of dystroglycan in the nervous system, particularly regarding its putative functions in neurons of the olfactory bulb, thalamus, hypothalamus and brainstem (Górecki et al., 1994; Zaccaria et al., 2001). Further use of inducible *Dag1* knockout models and precise domain deletions is crucial to expand our understanding of the specific roles for αDG and βDG in both developing and mature cell types. Additional factors are possibly at play in the dystroglycan-deficient brain that are not discussed in this Review, including aberrant energy metabolism and cholinergic signaling (Rae et al., 1998, 2002; Takeuchi et al., 2009; Tuon et al., 2010; Parames et al., 2014). In future work, parsing the relationship between these pathologic elements should clarify the functional roles of dystroglycan and elucidate the order of events in its dysfunction. Continued study and development of clinically relevant animal models will be a key endeavor in understanding the basic functions of αDG in the nervous system and in designing rational therapies for α-dystroglycanopathies.
